# Patient and Caregiver Symptom Trajectory: The Last 2 Months of Cancer Home Hospice

**DOI:** 10.1093/geroni/igab046.2939

**Published:** 2021-12-17

**Authors:** Maija Reblin, Eli Iacob, Djin Tay, Miranda Jones, Kristin Cloyes, Anna Beck, Kathi Mooney, Lee Ellington

**Affiliations:** 1 Moffitt Cancer Center, Tampa, Florida, United States; 2 University of Utah, Salt Lake City, Utah, United States; 3 University of Utah, Portland, Oregon, United States; 4 Huntsman Cancer Institute, Salt Lake City, Utah, United States

## Abstract

Patient symptom management is a fundamental goal of cancer home hospice care. However, informal family caregivers, who are primarily responsible for daily patient care, also experience negative symptoms, especially at the end of the patient’s life. While research has attended to patient symptom progression in home hospice, little research focuses on caregiver symptoms. To address this, we examined the frequency of both patient and caregiver symptoms to determine how these symptoms change in the last two months of the patient’s life. Sixty-three cancer hospice caregivers from 4 US states prospectively reported daily patient and caregiver symptoms via an Interactive Voice Response phone system. We analyzed data from up to the last 60 days of the patient’s life. Most caregivers were female (71.4%), Caucasian (88.9%), spouses of the patient (46%); average age was 59 years old (SD=13). Patients were mostly female (54%), with diverse solid tumor cancer diagnoses, and 72 years old (SD=11) on average. Most commonly reported moderate-to-severe patient symptoms were fatigue (67%), pain (47.5%), and loss in appetite (42.3%). Most common moderate-to-severe caregiver symptoms were fatigue (57.8%), trouble sleeping (45.1%), anxiety (52%), and depression (40.4%). Patient and caregiver symptoms were significantly correlated (Pearson r = .51, p<.001). Mixed-effects models found that both patient and caregiver symptoms (collapsed by week) worsened as patient death approached (ps <.01). Researchers and clinicians who are aware of the strong relationship between patient and caregiver symptoms are best able to address caregiver symptoms as part of hospice care, particularly as patient death approaches.

